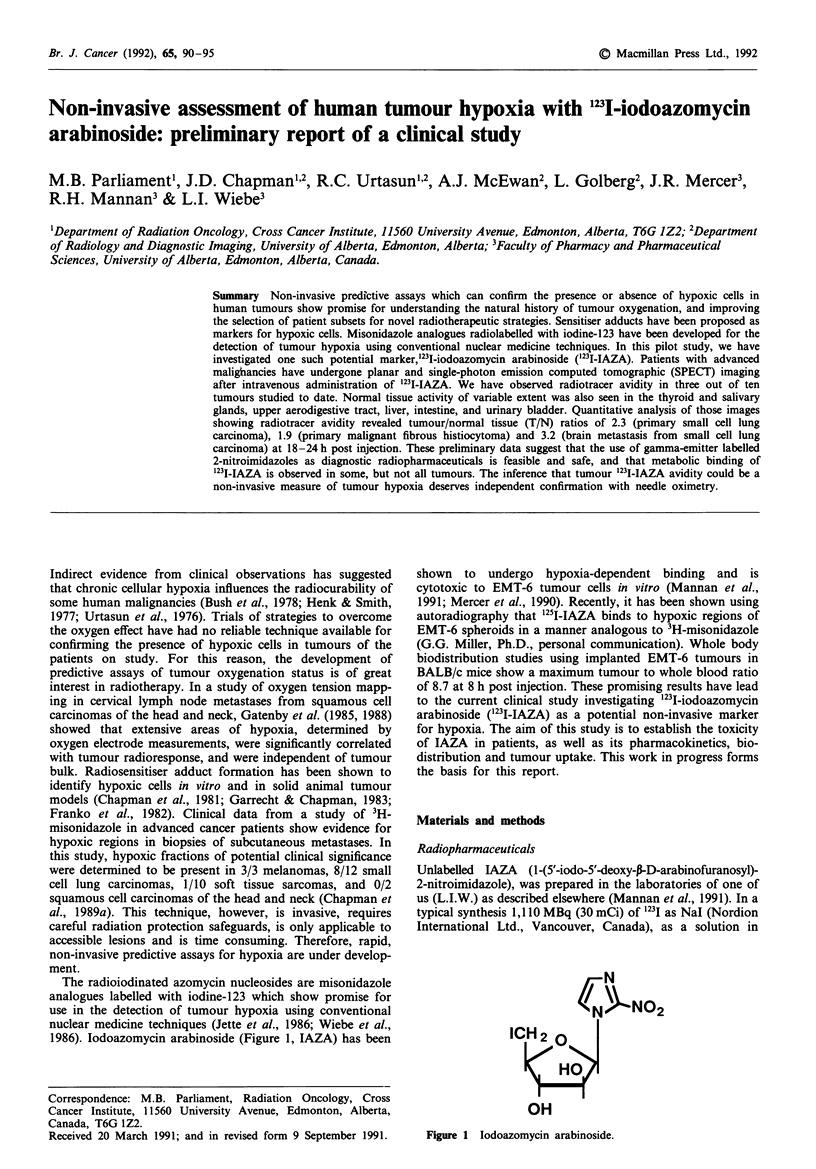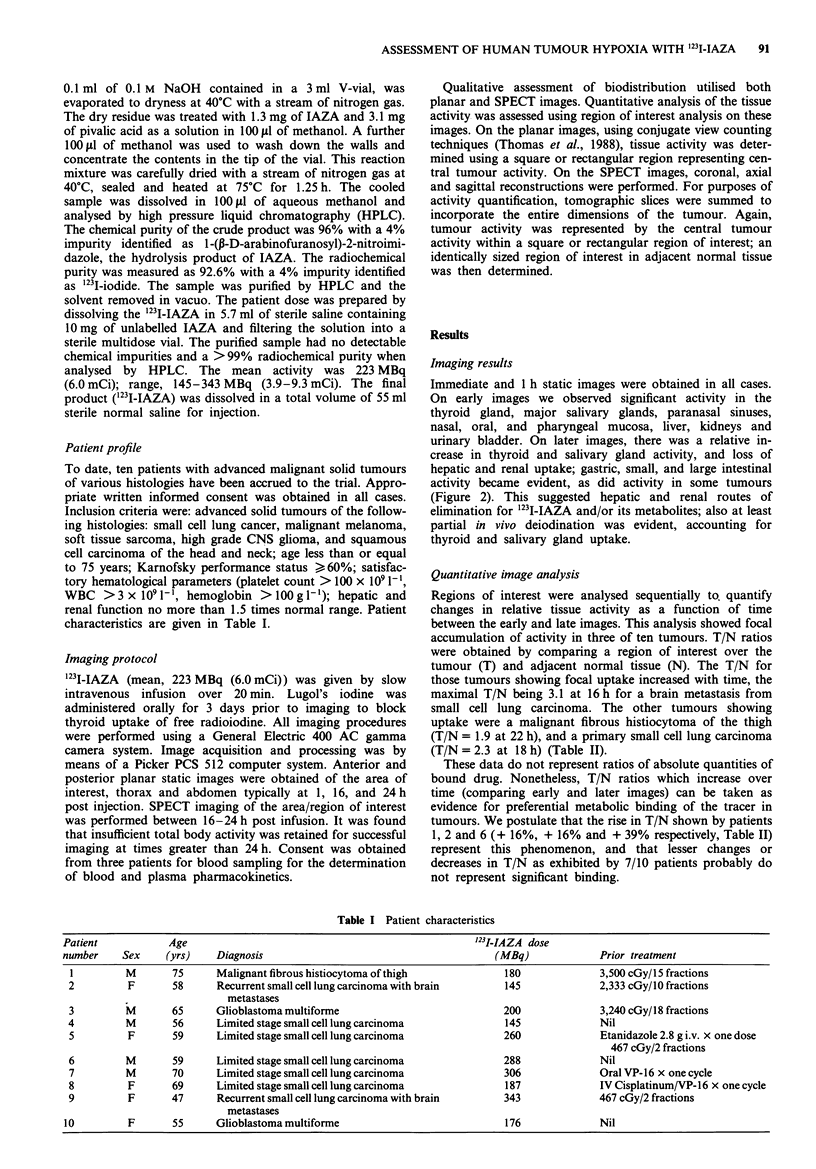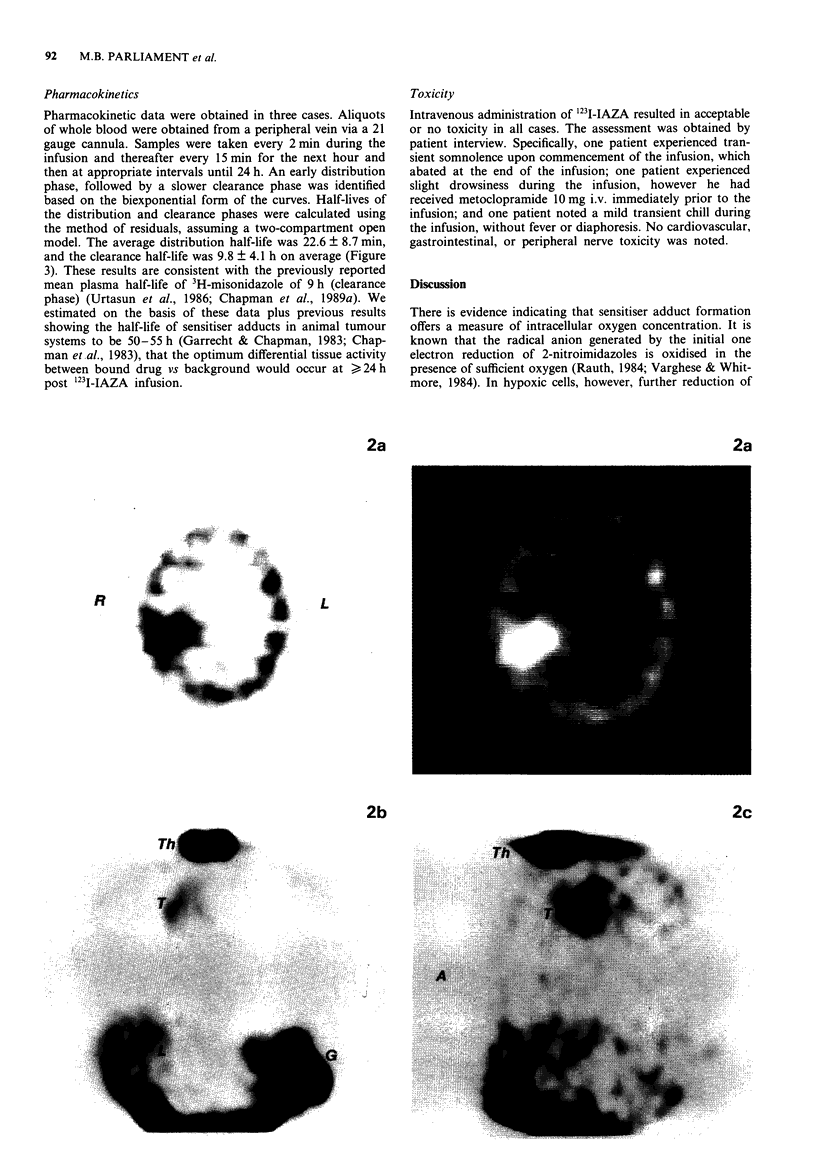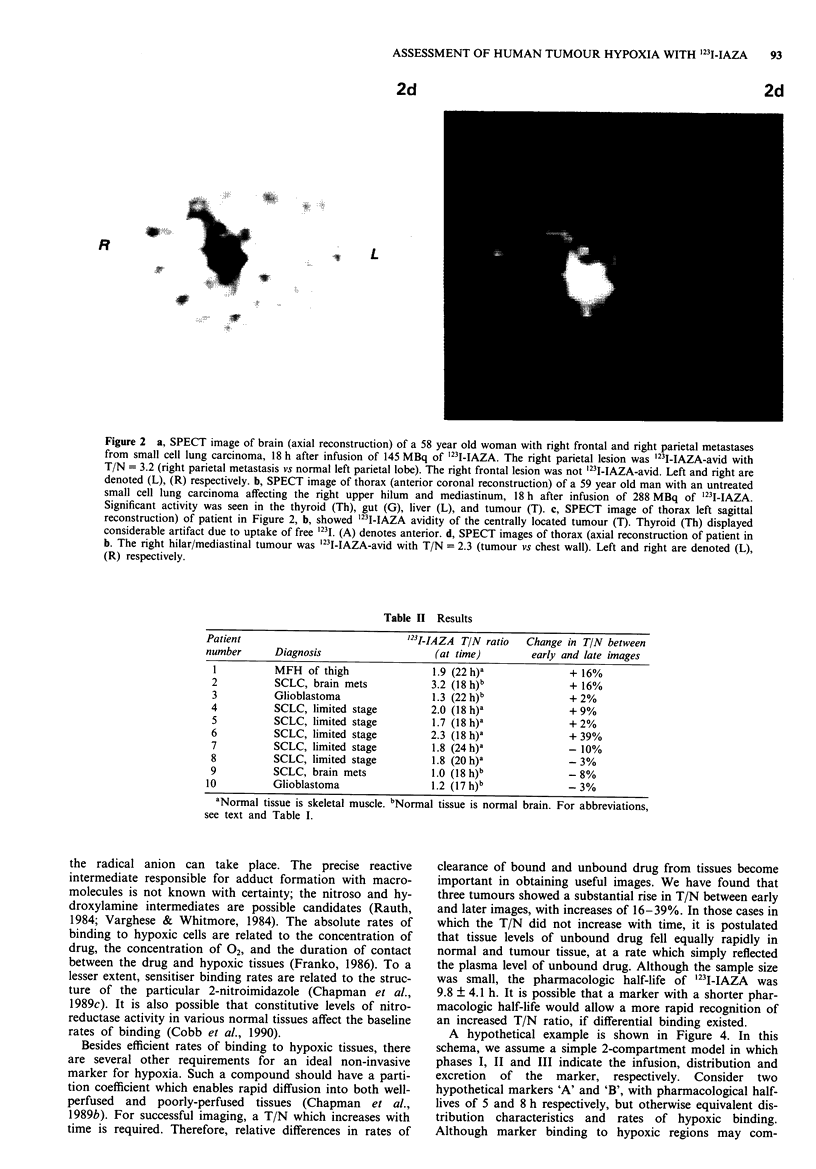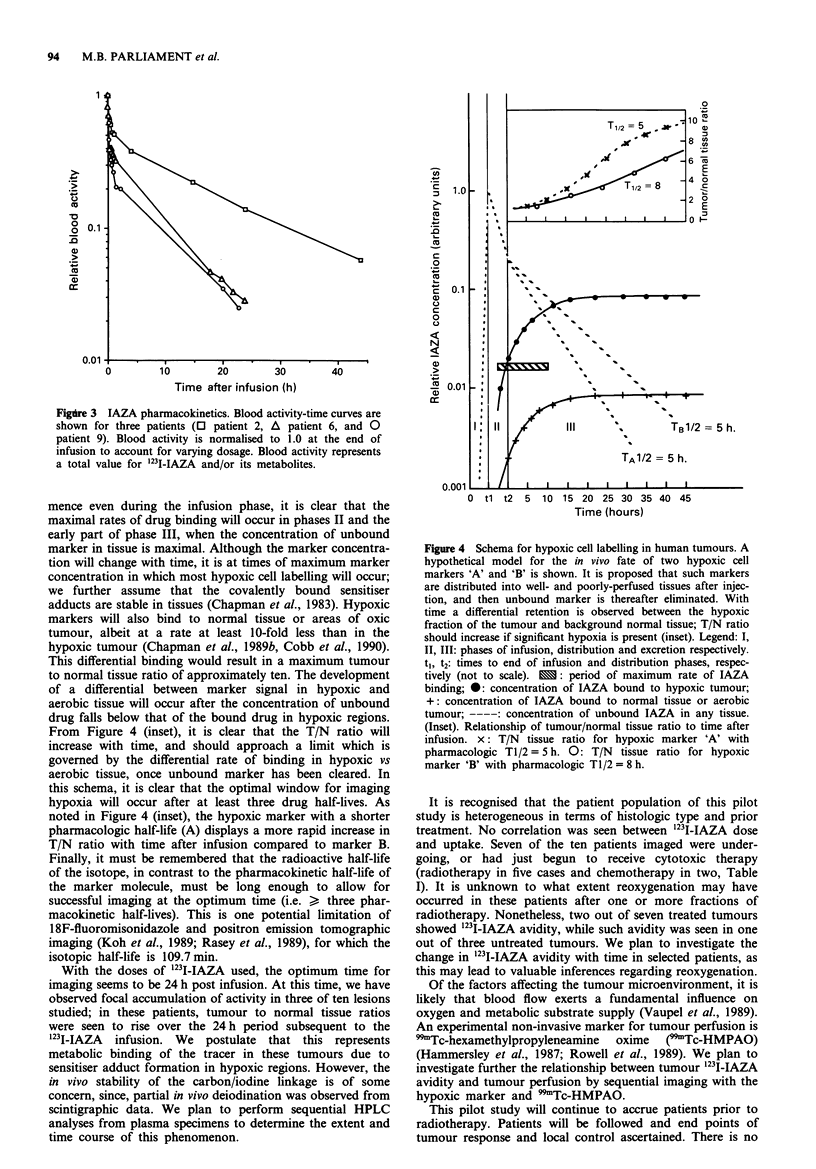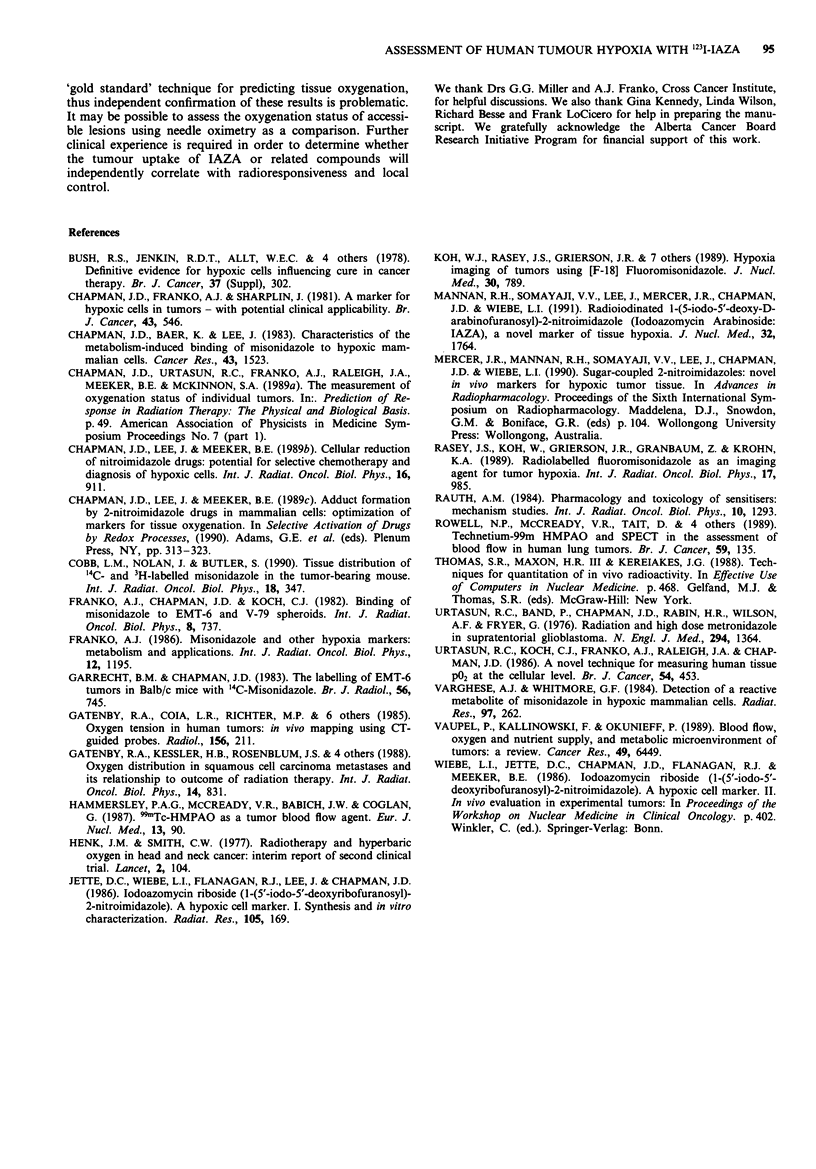# Non-invasive assessment of human tumour hypoxia with 123I-iodoazomycin arabinoside: preliminary report of a clinical study.

**DOI:** 10.1038/bjc.1992.17

**Published:** 1992-01

**Authors:** M. B. Parliament, J. D. Chapman, R. C. Urtasun, A. J. McEwan, L. Golberg, J. R. Mercer, R. H. Mannan, L. I. Wiebe

**Affiliations:** Department of Radiation Oncology, Cross Cancer Institute, Edmonton, Alberta, Canada.

## Abstract

**Images:**


*Br. J. Cancer* (1992), **65**, 90–95
© Macmillan Press Ltd., 1992

### Non-invasive assessment of human tumour hypoxia with $^{123}$I-iodoazomycin arabinoside: preliminary report of a clinical study

M.B. Parliament[1], J.D. Chapman[1,2], R.C. Urtasun[1,2], A.J. McEwan[2], L. Golberg[2], J.R. Mercer[3], R.H. Mannan[3] & L.I. Wiebe[3]

[1]*Department of Radiation Oncology, Cross Cancer Institute, 11560 University Avenue, Edmonton, Alberta, T6G 1Z2;* [2]*Department of Radiology and Diagnostic Imaging, University of Alberta, Edmonton, Alberta;* [3]*Faculty of Pharmacy and Pharmaceutical Sciences, University of Alberta, Edmonton, Alberta, Canada.*

**Summary** Non-invasive predictive assays which can confirm the presence or absence of hypoxic cells in human tumours show promise for understanding the natural history of tumour oxygenation, and improving the selection of patient subsets for novel radiotherapeutic strategies. Sensitiser adducts have been proposed as markers for hypoxic cells. Misonidazole analogues radiolabelled with iodine-123 have been developed for the detection of tumour hypoxia using conventional nuclear medicine techniques. In this pilot study, we have investigated one such potential marker, $^{123}$I-iodoazomycin arabinoside ($^{123}$I-IAZA). Patients with advanced malignancies have undergone planar and single-photon emission computed tomographic (SPECT) imaging after intravenous administration of $^{123}$I-IAZA. We have observed radiotracer avidity in three out of ten tumours studied to date. Normal tissue activity of variable extent was also seen in the thyroid and salivary glands, upper aerodigestive tract, liver, intestine, and urinary bladder. Quantitative analysis of those images showing radiotracer avidity revealed tumour/normal tissue (T/N) ratios of 2.3 (primary small cell lung carcinoma), 1.9 (primary malignant fibrous histiocytoma) and 3.2 (brain metastasis from small cell lung carcinoma) at 18–24 h post injection. These preliminary data suggest that the use of gamma-emitter labelled 2-nitroimidazoles as diagnostic radiopharmaceuticals is feasible and safe, and that metabolic binding of $^{123}$I-IAZA is observed in some, but not all tumours. The inference that tumour $^{123}$I-IAZA avidity could be a non-invasive measure of tumour hypoxia deserves independent confirmation with needle oximetry.

Indirect evidence from clinical observations has suggested that chronic cellular hypoxia influences the radiocurability of some human malignancies (Bush *et al.*, 1978; Henk & Smith, 1977; Urtasun *et al.*, 1976). Trials of strategies to overcome the oxygen effect have had no reliable technique available for confirming the presence of hypoxic cells in tumours of the patients on study. For this reason, the development of predictive assays of tumour oxygenation status is of great interest in radiotherapy. In a study of oxygen tension mapping in cervical lymph node metastases from squamous cell carcinomas of the head and neck, Gatenby *et al.* (1985, 1988) showed that extensive areas of hypoxia, determined by oxygen electrode measurements, were significantly correlated with tumour radioresponse, and were independent of tumour bulk. Radiosensitiser adduct formation has been shown to identify hypoxic cells *in vitro* and in solid animal tumour models (Chapman *et al.*, 1981; Garrecht & Chapman, 1983; Franko *et al.*, 1982). Clinical data from a study of $^{3}$H-misonidazole in advanced cancer patients show evidence for hypoxic regions in biopsies of subcutaneous metastases. In this study, hypoxic fractions of potential clinical significance were determined to be present in 3/3 melanomas, 8/12 small cell lung carcinomas, 1/10 soft tissue sarcomas, and 0/2 squamous cell carcinomas of the head and neck (Chapman *et al.*, 1989*a*). This technique, however, is invasive, requires careful radiation protection safeguards, is only applicable to accessible lesions and is time consuming. Therefore, rapid, non-invasive predictive assays for hypoxia are under development.

The radioiodinated azomycin nucleosides are misonidazole analogues labelled with iodine-123 which show promise for use in the detection of tumour hypoxia using conventional nuclear medicine techniques (Jette *et al.*, 1986; Wiebe *et al.*, 1986). Iodoazomycin arabinoside (Figure 1, IAZA) has been shown to undergo hypoxia-dependent binding and is cytotoxic to EMT-6 tumour cells *in vitro* (Mannan *et al.*, 1991; Mercer *et al.*, 1990). Recently, it has been shown using autoradiography that $^{125}$I-IAZA binds to hypoxic regions of EMT-6 spheroids in a manner analogous to $^{3}$H-misonidazole (G.G. Miller, Ph.D., personal communication). Whole body biodistribution studies using implanted EMT-6 tumours in BALB/c mice show a maximum tumour to whole blood ratio of 8.7 at 8 h post injection. These promising results have lead to the current clinical study investigating $^{123}$I-iodoazomycin arabinoside ($^{123}$I-IAZA) as a potential non-invasive marker for hypoxia. The aim of this study is to establish the toxicity of IAZA in patients, as well as its pharmacokinetics, biodistribution and tumour uptake. This work in progress forms the basis for this report.

### Materials and methods

### *Radiopharmaceuticals*

Unlabelled IAZA (1-(5′-iodo-5′-deoxy-β-D-arabinofuranosyl)-2-nitroimidazole), was prepared in the laboratories of one of us (L.I.W.) as described elsewhere (Mannan *et al.*, 1991). In a typical synthesis 1,110 MBq (30 mCi) of $^{123}$I as NaI (Nordion International Ltd., Vancouver, Canada), as a solution in

**Figure 1** Iodoazomycin arabinoside.

Correspondence: M.B. Parliament, Radiation Oncology, Cross Cancer Institute, 11560 University Avenue, Edmonton, Alberta, Canada, T6G 1Z2.
Received 20 March 1991; and in revised form 9 September 1991.

0.1 ml of 0.1 M NaOH contained in a 3 ml V-vial, was evaporated to dryness at 40°C with a stream of nitrogen gas. The dry residue was treated with 1.3 mg of IAZA and 3.1 mg of pivalic acid as a solution in 100 µl of methanol. A further 100 µl of methanol was used to wash down the walls and concentrate the contents in the tip of the vial. This reaction mixture was carefully dried with a stream of nitrogen gas at 40°C, sealed and heated at 75°C for 1.25 h. The cooled sample was dissolved in 100 µl of aqueous methanol and analysed by high pressure liquid chromatography (HPLC). The chemical purity of the crude product was 96% with a 4% impurity identified as 1-(β-D-arabinofuranosyl)-2-nitroimidazole, the hydrolysis product of IAZA. The radiochemical purity was measured as 92.6% with a 4% impurity identified as $^{123}$I-iodide. The sample was purified by HPLC and the solvent removed in vacuo. The patient dose was prepared by dissolving the $^{123}$I-IAZA in 5.7 ml of sterile saline containing 10 mg of unlabelled IAZA and filtering the solution into a sterile multidose vial. The purified sample had no detectable chemical impurities and a >99% radiochemical purity when analysed by HPLC. The mean activity was 223 MBq (6.0 mCi); range, 145–343 MBq (3.9–9.3 mCi). The final product ($^{123}$I-IAZA) was dissolved in a total volume of 55 ml sterile normal saline for injection.

### *Patient profile*

To date, ten patients with advanced malignant solid tumours of various histologies have been accrued to the trial. Appropriate written informed consent was obtained in all cases. Inclusion criteria were: advanced solid tumours of the following histologies: small cell lung cancer, malignant melanoma, soft tissue sarcoma, high grade CNS glioma, and squamous cell carcinoma of the head and neck; age less than or equal to 75 years; Karnofsky performance status ⩾60%; satisfactory hematological parameters (platelet count $> 100 \times 10^9 l^{-1}$, WBC $> 3 \times 10^9 l^{-1}$, hemoglobin $> 100 g l^{-1}$); hepatic and renal function no more than 1.5 times normal range. Patient characteristics are given in Table I.

### *Imaging protocol*

$^{123}$I-IAZA (mean, 223 MBq (6.0 mCi)) was given by slow intravenous infusion over 20 min. Lugol's iodine was administered orally for 3 days prior to imaging to block thyroid uptake of free radioiodine. All imaging procedures were performed using a General Electric 400 AC gamma camera system. Image acquisition and processing was by means of a Picker PCS 512 computer system. Anterior and posterior planar static images were obtained of the area of interest, thorax and abdomen typically at 1, 16, and 24 h post injection. SPECT imaging of the area/region of interest was performed between 16–24 h post infusion. It was found that insufficient total body activity was retained for successful imaging at times greater than 24 h. Consent was obtained from three patients for blood sampling for the determination of blood and plasma pharmacokinetics.

Qualitative assessment of biodistribution utilised both planar and SPECT images. Quantitative analysis of the tissue activity was assessed using region of interest analysis on these images. On the planar images, using conjugate view counting techniques (Thomas *et al.*, 1988), tissue activity was determined using a square or rectangular region representing central tumour activity. On the SPECT images, coronal, axial and sagittal reconstructions were performed. For purposes of activity quantification, tomographic slices were summed to incorporate the entire dimensions of the tumour. Again, tumour activity was represented by the central tumour activity within a square or rectangular region of interest; an identically sized region of interest in adjacent normal tissue was then determined.

### Results

### *Imaging results*

Immediate and 1 h static images were obtained in all cases. On early images we observed significant activity in the thyroid gland, major salivary glands, paranasal sinuses, nasal, oral, and pharyngeal mucosa, liver, kidneys and urinary bladder. On later images, there was a relative increase in thyroid and salivary gland activity, and loss of hepatic and renal uptake; gastric, small, and large intestinal activity became evident, as did activity in some tumours (Figure 2). This suggested hepatic and renal routes of elimination for $^{123}$I-IAZA and/or its metabolites; also at least partial *in vivo* deiodination was evident, accounting for thyroid and salivary gland uptake.

### *Quantitative image analysis*

Regions of interest were analysed sequentially to quantify changes in relative tissue activity as a function of time between the early and late images. This analysis showed focal accumulation of activity in three of ten tumours. T/N ratios were obtained by comparing a region of interest over the tumour (T) and adjacent normal tissue (N). The T/N for those tumours showing focal uptake increased with time, the maximal T/N being 3.1 at 16 h for a brain metastasis from small cell lung carcinoma. The other tumours showing uptake were a malignant fibrous histiocytoma of the thigh (T/N = 1.9 at 22 h), and a primary small cell lung carcinoma (T/N = 2.3 at 18 h) (Table II).

These data do not represent ratios of absolute quantities of bound drug. Nonetheless, T/N ratios which increase over time (comparing early and later images) can be taken as evidence for preferential metabolic binding of the tracer in tumours. We postulate that the rise in T/N shown by patients 1, 2 and 6 (+ 16%, + 16% and + 39% respectively, Table II) represent this phenomenon, and that lesser changes or decreases in T/N as exhibited by 7/10 patients probably do not represent significant binding.

**Table I** Patient characteristics

| *Patient number* | *Sex* | *Age (yrs)* | *Diagnosis* | *$^{123}$I-IAZA dose (MBq)* | *Prior treatment* |
|---|---|---|---|---|---|
| 1 | M | 75 | Malignant fibrous histiocytoma of thigh | 180 | 3,500 cGy/15 fractions |
| 2 | F | 58 | Recurrent small cell lung carcinoma with brain metastases | 145 | 2,333 cGy/10 fractions |
| 3 | M | 65 | Glioblastoma multiforme | 200 | 3,240 cGy/18 fractions |
| 4 | M | 56 | Limited stage small cell lung carcinoma | 145 | Nil |
| 5 | F | 59 | Limited stage small cell lung carcinoma | 260 | Etanidazole 2.8 g i.v. × one dose 467 cGy/2 fractions |
| 6 | M | 59 | Limited stage small cell lung carcinoma | 288 | Nil |
| 7 | M | 70 | Limited stage small cell lung carcinoma | 306 | Oral VP-16 × one cycle |
| 8 | F | 69 | Limited stage small cell lung carcinoma | 187 | IV Cisplatinum/VP-16 × one cycle |
| 9 | F | 47 | Recurrent small cell lung carcinoma with brain metastases | 343 | 467 cGy/2 fractions |
| 10 | F | 55 | Glioblastoma multiforme | 176 | Nil |

*Pharmacokinetics*

Pharmacokinetic data were obtained in three cases. Aliquots of whole blood were obtained from a peripheral vein via a 21 gauge cannula. Samples were taken every 2 min during the infusion and thereafter every 15 min for the next hour and then at appropriate intervals until 24 h. An early distribution phase, followed by a slower clearance phase was identified based on the biexponential form of the curves. Half-lives of the distribution and clearance phases were calculated using the method of residuals, assuming a two-compartment open model. The average distribution half-life was 22.6 ± 8.7 min, and the clearance half-life was 9.8 ± 4.1 h on average (Figure 3). These results are consistent with the previously reported mean plasma half-life of $^{3}$H-misonidazole of 9 h (clearance phase) (Urtasun *et al.*, 1986; Chapman *et al.*, 1989*a*). We estimated on the basis of these data plus previous results showing the half-life of sensitiser adducts in animal tumour systems to be 50–55 h (Garrecht & Chapman, 1983; Chapman *et al.*, 1983), that the optimum differential tissue activity between bound drug *vs* background would occur at ⩾24 h post $^{123}$I-IAZA infusion.

*Toxicity*

Intravenous administration of $^{123}$I-IAZA resulted in acceptable or no toxicity in all cases. The assessment was obtained by patient interview. Specifically, one patient experienced transient somnolence upon commencement of the infusion, which abated at the end of the infusion; one patient experienced slight drowsiness during the infusion, however he had received metoclopramide 10 mg i.v. immediately prior to the infusion; and one patient noted a mild transient chill during the infusion, without fever or diaphoresis. No cardiovascular, gastrointestinal, or peripheral nerve toxicity was noted.

### Discussion

There is evidence indicating that sensitiser adduct formation offers a measure of intracellular oxygen concentration. It is known that the radical anion generated by the initial one electron reduction of 2-nitroimidazoles is oxidised in the presence of sufficient oxygen (Rauth, 1984; Varghese & Whitmore, 1984). In hypoxic cells, however, further reduction of

**Figure 2** **a**, SPECT image of brain (axial reconstruction) of a 58 year old woman with right frontal and right parietal metastases from small cell lung carcinoma, 18 h after infusion of 145 MBq of $^{123}$I-IAZA. The right parietal lesion was $^{123}$I-IAZA-avid with T/N = 3.2 (right parietal metastasis *vs* normal left parietal lobe). The right frontal lesion was not $^{123}$I-IAZA-avid. Left and right are denoted (L), (R) respectively. **b**, SPECT image of thorax (anterior coronal reconstruction) of a 59 year old man with an untreated small cell lung carcinoma affecting the right upper hilum and mediastinum, 18 h after infusion of 288 MBq of $^{123}$I-IAZA. Significant activity was seen in the thyroid (Th), gut (G), liver (L), and tumour (T). **c**, SPECT image of thorax left sagittal reconstruction) of patient in Figure 2, **b**, showed $^{123}$I-IAZA avidity of the centrally located tumour (T). Thyroid (Th) displayed considerable artifact due to uptake of free $^{123}$I. (A) denotes anterior. **d**, SPECT images of thorax (axial reconstruction of patient in **b**. The right hilar/mediastinal tumour was $^{123}$I-IAZA-avid with T/N = 2.3 (tumour *vs* chest wall). Left and right are denoted (L), (R) respectively.

**Table II** Results

| *Patient number* | *Diagnosis* | *$^{123}$I-IAZA T/N ratio (at time)* | *Change in T/N between early and late images* |
|---|---|---|---|
| 1 | MFH of thigh | 1.9 (22 h)[a] | + 16% |
| 2 | SCLC, brain mets | 3.2 (18 h)[b] | + 16% |
| 3 | Glioblastoma | 1.3 (22 h)[b] | + 2% |
| 4 | SCLC, limited stage | 2.0 (18 h)[a] | + 9% |
| 5 | SCLC, limited stage | 1.7 (18 h)[a] | + 2% |
| 6 | SCLC, limited stage | 2.3 (18 h)[a] | + 39% |
| 7 | SCLC, limited stage | 1.8 (24 h)[a] | − 10% |
| 8 | SCLC, limited stage | 1.8 (20 h)[a] | − 3% |
| 9 | SCLC, brain mets | 1.0 (18 h)[b] | − 8% |
| 10 | Glioblastoma | 1.2 (17 h)[b] | − 3% |

[a]Normal tissue is skeletal muscle. [b]Normal tissue is normal brain. For abbreviations, see text and Table I.

the radical anion can take place. The precise reactive intermediate responsible for adduct formation with macromolecules is not known with certainty; the nitroso and hydroxylamine intermediates are possible candidates (Rauth, 1984; Varghese & Whitmore, 1984). The absolute rates of binding to hypoxic cells are related to the concentration of drug, the concentration of $O_2$, and the duration of contact between the drug and hypoxic tissues (Franko, 1986). To a lesser extent, sensitiser binding rates are related to the structure of the particular 2-nitroimidazole (Chapman *et al.*, 1989*c*). It is also possible that constitutive levels of nitroreductase activity in various normal tissues affect the baseline rates of binding (Cobb *et al.*, 1990).

Besides efficient rates of binding to hypoxic tissues, there are several other requirements for an ideal non-invasive marker for hypoxia. Such a compound should have a partition coefficient which enables rapid diffusion into both well-perfused and poorly-perfused tissues (Chapman *et al.*, 1989*b*). For successful imaging, a T/N which increases with time is required. Therefore, relative differences in rates of clearance of bound and unbound drug from tissues become important in obtaining useful images. We have found that three tumours showed a substantial rise in T/N between early and later images, with increases of 16–39%. In those cases in which the T/N did not increase with time, it is postulated that tissue levels of unbound drug fell equally rapidly in normal and tumour tissue, at a rate which simply reflected the plasma level of unbound drug. Although the sample size was small, the pharmacologic half-life of $^{123}$I-IAZA was 9.8 ± 4.1 h. It is possible that a marker with a shorter pharmacologic half-life would allow a more rapid recognition of an increased T/N ratio, if differential binding existed.

A hypothetical example is shown in Figure 4. In this schema, we assume a simple 2-compartment model in which phases I, II and III indicate the infusion, distribution and excretion of the marker, respectively. Consider two hypothetical markers 'A' and 'B', with pharmacological half-lives of 5 and 8 h respectively, but otherwise equivalent distribution characteristics and rates of hypoxic binding. Although marker binding to hypoxic regions may com-

Figure 3 IAZA pharmacokinetics. Blood activity-time curves are shown for three patients (□ patient 2, Δ patient 6, and O patient 9). Blood activity is normalised to 1.0 at the end of infusion to account for varying dosage. Blood activity represents a total value for $^{123}$I-IAZA and/or its metabolites.

mence even during the infusion phase, it is clear that the maximal rates of drug binding will occur in phases II and the early part of phase III, when the concentration of unbound marker in tissue is maximal. Although the marker concentration will change with time, it is at times of maximum marker concentration in which most hypoxic cell labelling will occur; we further assume that the covalently bound sensitiser adducts are stable in tissues (Chapman et al., 1983). Hypoxic markers will also bind to normal tissue or areas of oxic tumour, albeit at a rate at least 10-fold less than in the hypoxic tumour (Chapman et al., 1989b, Cobb et al., 1990). This differential binding would result in a maximum tumour to normal tissue ratio of approximately ten. The development of a differential between marker signal in hypoxic and aerobic tissue will occur after the concentration of unbound drug falls below that of the bound drug in hypoxic regions. From Figure 4 (inset), it is clear that the T/N ratio will increase with time, and should approach a limit which is governed by the differential rate of binding in hypoxic *vs* aerobic tissue, once unbound marker has been cleared. In this schema, it is clear that the optimal window for imaging hypoxia will occur after at least three drug half-lives. As noted in Figure 4 (inset), the hypoxic marker with a shorter pharmacologic half-life (A) displays a more rapid increase in T/N ratio with time after infusion compared to marker B. Finally, it must be remembered that the radioactive half-life of the isotope, in contrast to the pharmacokinetic half-life of the marker molecule, must be long enough to allow for successful imaging at the optimum time (i.e. $\geqslant$ three pharmacokinetic half-lives). This is one potential limitation of 18F-fluoromisonidazole and positron emission tomographic imaging (Koh et al., 1989; Rasey et al., 1989), for which the isotopic half-life is 109.7 min.

With the doses of $^{123}$I-IAZA used, the optimum time for imaging seems to be 24 h post infusion. At this time, we have observed focal accumulation of activity in three of ten lesions studied; in these patients, tumour to normal tissue ratios were seen to rise over the 24 h period subsequent to the $^{123}$I-IAZA infusion. We postulate that this represents metabolic binding of the tracer in these tumours due to sensitiser adduct formation in hypoxic regions. However, the *in vivo* stability of the carbon/iodine linkage is of some concern, since, partial *in vivo* deiodination was observed from scintigraphic data. We plan to perform sequential HPLC analyses from plasma specimens to determine the extent and time course of this phenomenon.

Figure 4 Schema for hypoxic cell labelling in human tumours. A hypothetical model for the *in vivo* fate of two hypoxic cell markers 'A' and 'B' is shown. It is proposed that such markers are distributed into well- and poorly-perfused tissues after injection, and then unbound marker is thereafter eliminated. With time a differential retention is observed between the hypoxic fraction of the tumour and background normal tissue; T/N ratio should increase if significant hypoxia is present (inset). Legend: I, II, III: phases of infusion, distribution and excretion respectively. $t_1$, $t_2$: times to end of infusion and distribution phases, respectively (not to scale). ▧: period of maximum rate of IAZA binding; ●: concentration of IAZA bound to hypoxic tumour; +: concentration of IAZA bound to normal tissue or aerobic tumour; ----: concentration of unbound IAZA in any tissue. (Inset). Relationship of tumour/normal tissue ratio to time after infusion. ×: T/N tissue ratio for hypoxic marker 'A' with pharmacologic T1/2 = 5 h. O: T/N tissue ratio for hypoxic marker 'B' with pharmacologic T1/2 = 8 h.

It is recognised that the patient population of this pilot study is heterogeneous in terms of histologic type and prior treatment. No correlation was seen between $^{123}$I-IAZA dose and uptake. Seven of the ten patients imaged were undergoing, or had just begun to receive cytotoxic therapy (radiotherapy in five cases and chemotherapy in two, Table I). It is unknown to what extent reoxygenation may have occurred in these patients after one or more fractions of radiotherapy. Nonetheless, two out of seven treated tumours showed $^{123}$I-IAZA avidity, while such avidity was seen in one out of three untreated tumours. We plan to investigate the change in $^{123}$I-IAZA avidity with time in selected patients, as this may lead to valuable inferences regarding reoxygenation.

Of the factors affecting the tumour microenvironment, it is likely that blood flow exerts a fundamental influence on oxygen and metabolic substrate supply (Vaupel et al., 1989). An experimental non-invasive marker for tumour perfusion is $^{99m}$Tc-hexamethylpropyleneamine oxime ($^{99m}$Tc-HMPAO) (Hammersley et al., 1987; Rowell et al., 1989). We plan to investigate further the relationship between tumour $^{123}$I-IAZA avidity and tumour perfusion by sequential imaging with the hypoxic marker and $^{99m}$Tc-HMPAO.

This pilot study will continue to accrue patients prior to radiotherapy. Patients will be followed and end points of tumour response and local control ascertained. There is no

'gold standard' technique for predicting tissue oxygenation, thus independent confirmation of these results is problematic. It may be possible to assess the oxygenation status of accessible lesions using needle oximetry as a comparison. Further clinical experience is required in order to determine whether the tumour uptake of IAZA or related compounds will independently correlate with radioresponsiveness and local control.

We thank Drs G.G. Miller and A.J. Franko, Cross Cancer Institute, for helpful discussions. We also thank Gina Kennedy, Linda Wilson, Richard Besse and Frank LoCicero for help in preparing the manuscript. We gratefully acknowledge the Alberta Cancer Board Research Initiative Program for financial support of this work.

**References**

BUSH, R.S., JENKIN, R.D.T., ALLT, W.E.C. & 4 others (1978). Definitive evidence for hypoxic cells influencing cure in cancer therapy. *Br. J. Cancer*, **37** (Suppl), 302.

CHAPMAN, J.D., FRANKO, A.J. & SHARPLIN, J. (1981). A marker for hypoxic cells in tumors – with potential clinical applicability. *Br. J. Cancer*, **43**, 546.

CHAPMAN, J.D., BAER, K. & LEE, J. (1983). Characteristics of the metabolism-induced binding of misonidazole to hypoxic mammalian cells. *Cancer Res.*, **43**, 1523.

CHAPMAN, J.D., URTASUN, R.C., FRANKO, A.J., RALEIGH, J.A., MEEKER, B.E. & MCKINNON, S.A. (1989*a*). The measurement of oxygenation status of individual tumors. In:. *Prediction of Response in Radiation Therapy: The Physical and Biological Basis*. p. 49. American Association of Physicists in Medicine Symposium Proceedings No. 7 (part 1).

CHAPMAN, J.D., LEE, J. & MEEKER, B.E. (1989*b*). Cellular reduction of nitroimidazole drugs: potential for selective chemotherapy and diagnosis of hypoxic cells. *Int. J. Radiat. Oncol. Biol. Phys.*, **16**, 911.

CHAPMAN, J.D., LEE, J. & MEEKER, B.E. (1989*c*). Adduct formation by 2-nitroimidazole drugs in mammalian cells: optimization of markers for tissue oxygenation. In *Selective Activation of Drugs by Redox Processes*, (1990). Adams, G.E. *et al.* (eds). Plenum Press, NY, pp. 313–323.

COBB, L.M., NOLAN, J. & BUTLER, S. (1990). Tissue distribution of $^{14}$C- and $^{3}$H-labelled misonidazole in the tumor-bearing mouse. *Int. J. Radiat. Oncol. Biol. Phys.*, **18**, 347.

FRANKO, A.J., CHAPMAN, J.D. & KOCH, C.J. (1982). Binding of misonidazole to EMT-6 and V-79 spheroids. *Int. J. Radiat. Oncol. Biol. Phys.*, **8**, 737.

FRANKO, A.J. (1986). Misonidazole and other hypoxia markers: metabolism and applications. *Int. J. Radiat. Oncol. Biol. Phys.*, **12**, 1195.

GARRECHT, B.M. & CHAPMAN, J.D. (1983). The labelling of EMT-6 tumors in Balb/c mice with $^{14}$C-Misonidazole. *Br. J. Radiol.*, **56**, 745.

GATENBY, R.A., COIA, L.R., RICHTER, M.P. & 6 others (1985). Oxygen tension in human tumors: *in vivo* mapping using CT-guided probes. *Radiol.*, **156**, 211.

GATENBY, R.A., KESSLER, H.B., ROSENBLUM, J.S. & 4 others (1988). Oxygen distribution in squamous cell carcinoma metastases and its relationship to outcome of radiation therapy. *Int. J. Radiat. Oncol. Biol. Phys.*, **14**, 831.

HAMMERSLEY, P.A.G., MCCREADY, V.R., BABICH, J.W. & COGLAN, G. (1987). $^{99m}$Tc-HMPAO as a tumor blood flow agent. *Eur. J. Nucl. Med.*, **13**, 90.

HENK, J.M. & SMITH, C.W. (1977). Radiotherapy and hyperbaric oxygen in head and neck cancer: interim report of second clinical trial. *Lancet*, **2**, 104.

JETTE, D.C., WIEBE, L.I., FLANAGAN, R.J., LEE, J. & CHAPMAN, J.D. (1986). Iodoazomycin riboside (1-(5′-iodo-5′-deoxyribofuranosyl)-2-nitroimidazole). A hypoxic cell marker. I. Synthesis and *in vitro* characterization. *Radiat. Res.*, **105**, 169.

KOH, W.J., RASEY, J.S., GRIERSON, J.R. & 7 others (1989). Hypoxia imaging of tumors using [F-18] Fluoromisonidazole. *J. Nucl. Med.*, **30**, 789.

MANNAN, R.H., SOMAYAJI, V.V., LEE, J., MERCER, J.R., CHAPMAN, J.D. & WIEBE, L.I. (1991). Radioiodinated 1-(5-iodo-5′-deoxy-D-arabinofuranosyl)-2-nitroimidazole (Iodoazomycin Arabinoside: IAZA), a novel marker of tissue hypoxia. *J. Nucl. Med.*, **32**, 1764.

MERCER, J.R., MANNAN, R.H., SOMAYAJI, V.V., LEE, J., CHAPMAN, J.D. & WIEBE, L.I. (1990). Sugar-coupled 2-nitroimidazoles: novel *in vivo* markers for hypoxic tumor tissue. In *Advances in Radiopharmacology*. Proceedings of the Sixth International Symposium on Radiopharmacology. Maddelena, D.J., Snowdon, G.M. & Boniface, G.R. (eds) p. 104. Wollongong University Press: Wollongong, Australia.

RASEY, J.S., KOH, W., GRIERSON, J.R., GRANBAUM, Z. & KROHN, K.A. (1989). Radiolabelled fluoromisonidazole as an imaging agent for tumor hypoxia. *Int. J. Radiat. Oncol. Biol. Phys.*, **17**, 985.

RAUTH, A.M. (1984). Pharmacology and toxicology of sensitisers: mechanism studies. *Int. J. Radiat. Oncol. Biol. Phys.*, **10**, 1293.

ROWELL, N.P., MCCREADY, V.R., TAIT, D. & 4 others (1989). Technetium-99m HMPAO and SPECT in the assessment of blood flow in human lung tumors. *Br. J. Cancer*, **59**, 135.

THOMAS, S.R., MAXON, H.R. III & KEREIAKES, J.G. (1988). Techniques for quantitation of in vivo radioactivity. In *Effective Use of Computers in Nuclear Medicine*. p. 468. Gelfand, M.J. & Thomas, S.R. (eds). McGraw-Hill: New York.

URTASUN, R.C., BAND, P., CHAPMAN, J.D., RABIN, H.R., WILSON, A.F. & FRYER, G. (1976). Radiation and high dose metronidazole in supratentorial glioblastoma. *N. Engl. J. Med.*, **294**, 1364.

URTASUN, R.C., KOCH, C.J., FRANKO, A.J., RALEIGH, J.A. & CHAPMAN, J.D. (1986). A novel technique for measuring human tissue $pO_2$ at the cellular level. *Br. J. Cancer*, **54**, 453.

VARGHESE, A.J. & WHITMORE, G.F. (1984). Detection of a reactive metabolite of misonidazole in hypoxic mammalian cells. *Radiat. Res.*, **97**, 262.

VAUPEL, P., KALLINOWSKI, F. & OKUNIEFF, P. (1989). Blood flow, oxygen and nutrient supply, and metabolic microenvironment of tumors: a review. *Cancer Res.*, **49**, 6449.

WIEBE, L.I., JETTE, D.C., CHAPMAN, J.D., FLANAGAN, R.J. & MEEKER, B.E. (1986). Iodoazomycin riboside (1-(5′-iodo-5′-deoxyribofuranosyl)-2-nitroimidazole). A hypoxic cell marker. II. *In vivo* evaluation in experimental tumors: In *Proceedings of the Workshop on Nuclear Medicine in Clinical Oncology*. p. 402. Winkler, C. (ed.). Springer-Verlag: Bonn.